# Efficient multiplexed genome engineering with a polycistronic tRNA and CRISPR guide-RNA reveals an important role of detonator in reproduction of *Drosophila melanogaster*

**DOI:** 10.1371/journal.pone.0245454

**Published:** 2021-01-14

**Authors:** Cristin Chon, Grace Chon, Yurika Matsui, Huiqing Zeng, Zhi-Chun Lai, Aimin Liu

**Affiliations:** Department of Biology, Eberly College of Science, Centers for Cellular Dynamics and Cellular and Molecular Investigation of Neurological Diseases, Huck Institutes of Life Sciences, The Pennsylvania State University, State College, PA, United States of America; University of British Columbia, CANADA

## Abstract

Genome association studies in human and genetic studies in mouse implicated members of the transmembrane protein 132 (TMEM132) family in multiple conditions including panic disorder, hearing loss, limb and kidney malformation. However, the presence of five TMEM132 paralogs in mammalian genomes makes it extremely challenging to reveal the full requirement for these proteins in vivo. In contrast, there is only one TMEM132 homolog, *detonator* (*dtn*), in the genome of fruit fly *Drosophila melanogaster*, enabling straightforward research into its *in vivo* function. In the current study, we generate multiple loss-of-function *dtn* mutant fly strains through a polycistronic tRNA-gRNA approach, and show that most embryos lacking both maternal and paternal dtn fail to hatch into larvae, indicating an essential role of *dtn* in *Drosophila* reproduction.

## Introduction

Our understanding of the molecular mechanisms underlying normal development and physiological function of animals including humans is crucial for developing strategies to prevent birth defects and treating diseases with genetic components such as autism and autoimmune diseases. A variety of approaches have made significant contributions toward this goal. One classic approach is the use of animal models in which individual gene function is removed, leading to structural and/or functional changes that lend insights in the pathology of human diseases (e.g. [1]). Historically, the house mouse has been the model of choice because it is a mammalian model, with the availability of advanced genome engineering tools such as embryonic stem cells and gene-targeting technologies [2]. However, the high cost of maintaining a large mouse colony, the long generation time and functional redundancy between paralogues, have limited the use of this model to fully understand the function of gene families with multiple members. Invertebrate models such as *Drosophila melanogaster* and *C*. *elegans* are preferred in many cases due to lack of gene redundancy, ease to scale up for genetic analyses and the availability of many genetic tools. However, the precise alterations of individual genes through reverse genetics have been difficult in these models until the new clustered regulatory palindromic interspaced short repeats (CRISPR)/CrRNA associated protein 9 (Cas9)-based mutagenesis approach revolutionized genetic manipulations in many model animals [3].

Another approach that has been instrumental for the identification of genes involved in disease pathogenesis is the association studies linking human diseases to specific genomic loci [4]. Such genome-wide association studies have recently implicated four out of five members of the transmembrane 132 (TMEM132) family of single-pass membrane proteins in a plethora of human disease conditions. *TMEM132B* and *TMEM132D* have been linked to intracranial aneurysms and anxiety/panic disorders, respectively [5–11]. With probable involvement in colorectal cancer, *TMEM132C* was among one of four genes of a cancer-associated locus [12]. A missense mutation in *TMEM132E* causes autosomal-recessive non-syndromic hearing loss [13]. Although *TMEM132A* has not been associated with human diseases, a recent study in mice suggested that loss of *Tmem132a* resulted in preweaning lethality and defects in multiple tissues including the neural tube, limbs and kidneys [14]. These studies indicate that the TMEM132 family of proteins plays critical roles in the development and function of multiple systems.

The potential functional redundancy among five TMEM132 family members in mammals presents a technical challenge as a quintuple mutant animal is needed to reveal the full requirement for these proteins, which is extremely difficult, time and labor intensive. In contrast, the fruit fly *Drosophila melanogaster* genome contains only one TMEM132 homologue named *detonator (dtn)*. *Dtn* was identified as a gene with enriched expression in nociceptors, and RNAi knockdown in nociceptors appeared to increase the larvae’s sensitivity to thermal stimulus [15]. However, the efficiency and potential off-target effect of this RNAi knockdown was not determined, making the roles of *dtn* in nociception uncertain. Moreover, the roles of *dtn* in other processes have not been determined.

CRISPR/Cas9-based genome engineering has enabled precise manipulation of the genomes of many animal species, including *Drosophila* [16]. One advantage of this approach is the possibility of altering multiple targets at the same time. Such multiplex engineering can be accomplished by introducing multiple guide RNAs (gRNAs) simultaneously in the forms of in vitro synthesized RNA or plasmids expressing individual gRNAs [17, 18]. In theory, multiple expression cassettes can be cloned into a single plasmid, although our unpublished results indicated that the construction of such plasmids could be challenging. A recent strategy took advantage of the efficient processing of transfer RNA (tRNA) to express a chimerical RNA comprising alternating tRNAs and gRNAs, targeting up to 16 loci at the same time in rice, human cells and in *Drosophila*, greatly simplifying multiplex engineering [19–21].

In this work, we targeted the 4^th^ and 11^th^ exons of *dtn* simultaneously in *Drosophila* germline with the tRNA-gRNA chimeric strategy. We achieved efficient deletion between the two targets, along with more frequent non-homology end-joining (NHEJ)-induced mutations at individual targets leading to frameshift and presumed protein truncations. Meanwhile, no mutation was detected in potential off-target loci, suggesting the high specificity of our approach. The resulting flies carrying mutant alleles of *dtn* were viable with no obvious structural defects, although *dtn/dtn* homozygous female flies were underrepresented. The hemizygous *dtn/Y* mutant males were fertile, but the *dtn/dtn* homozygous mutant females laid fewer eggs than heterozygous flies. Furthermore, most eggs lacking maternal and zygotic dtn failed to complete embryonic development and hatch. Finally, similar defects in reproduction were observed by crossing *dtn* mutant flies to flies with deficiency around the *dtn* locus. These results suggest that *dtn* plays an important role in *Drosophila* reproduction.

## Materials and methods

### Construction and injection of the polycistronic tRNA-gRNA plasmid targeting dtn

We PCR amplified two tRNA-gRNA fragments using primers listed in Table 1, and pGTR (Addgene #63143) as template, and cloned them into linearized pU6-Bbs1-chiRNA backbone (Addgene #45946; also amplified through PCR) with Gibson assembly (New England Biolabs, #E5510S). After confirming the sequence of the assembled plasmid, we sent it to Best Gene Inc (www.thebestgene.com, Chino Hills, CA) for injection into *Drosophila* stock BDSC#51324, which expresses vas-Cas9 from chromosome III.

**Table 1:** PCR reactions for the construction of pU6-dtn-gRNA plasmid.

PCR1	L5F: TATGT TTTCC TCAAT ACTTC -gcaacaaagcaccagtgg	Template: pGTR
	gRNA[ex4]-R: 5’ GCAGCTCTGTTTATGTGGAT -tgcaccagccggg-3’	
PCR2	gRNA[*ex4*]-F: 5’ ATCCACATAAACAGAGCTGC -gttttagagctagaa-3’ gRNA[*ex11*]-R: 5’ -GTTCGATTCGCTGACACCAA -tgcaccagccggg-3’	pGTR
PCR3	gRNA[*ex11*]-F: 5’- TTGGT GTCAG CGAAT CGAAC—GTTTT AGAGC TAGAA ATAG -3’	pU6-Bbs1-chiRNA
	pU6R: GAAGT ATTGA GGAAA ACATA	

### Fly stocks

All fruit flies were cultured with food containing yeast (2.5% w/v), soy flour (1% w/v), cornmeal (7.3% w/v), corn syrup (7.7% v/v), agar (0.57% w/v), propionic acid (0.48% v/v) and tegosept (0.16% w/v, pre-dissolved in ethanol at 10% w/v). X-chromosome balancer lines used were Dmel\Binsinscy (flybase ID: FBba0000019) and Dmel\FM7a (flybase ID: FBba0000007). The two deficiency lines used were 7712 (Df(1)Exel6238,w[118]P{2{+mC} = XP-U}Exel6238/FM7c) and 25730 (Df(1)BSC640,w[118]Binsinscy).

### Fly lysis and PCR genotyping

When wandering larvae and/or pupae were present in the vial, we anesthetized the parental flies with CO_2_, euthanized them at –80°C for at least 30 min. Subsequently, we lysed each fly in 30μl lysis buffer (10 μg/ml proteinase K in 10 mM TrisHCl, pH8.8, 0.1% Tween 20). We performed PCR genotyping reactions using primers listed in Table 2 in an Eppendorf or BioRad thermocycler. When needed, PstI or TaqI was added to half of the reaction upon completion of PCR and incubated at a proper temperature for 3 hrs.

**Table 2:** *dtn* mutant screening strategy.

	Primers	PCR product	Restriction digest
Exon 4 NHEJ	R2 GAAGCGACTAATTGCCAAGC	394bp	Cut with PstI
			wt: 287bp + 106bp
			NHEJ: 394 bp
	R3 GGCATCCAAACGATGAACTT		
Exon 11 NHEJ	R4 GCCATAGCCATAGCCACAAC	382bp	Cut with TaqI
			wt: 165bp + 216bp
			NHEJ: 382bp
	R5 AATCTCGTCCGTTCCATTTG		
Exon 4–11 Deletion	R2 GAAGCGACTAATTGCCAAGC	WT: 3045bp	Cut with NheI would give rise to three products: 242bp + 154bp + 108bp
		Deletion: 503bp	
	R5 AATCTCGTCCGTTCCATTTG		

### Egg hatching assay

10~15 adult female and 3~5 adult male flies were cultured with yeast paste supplement for 3 days, after which the female flies were allowed to lay eggs on apple juice dishes. Eggs were collected several times over a 24-hr. period, and allowed to hatch for 48 hrs. Unhatched eggs were dechorinated with 50% bleach and fixed in 4% paraformaldehyde briefly. To visualize the cell nuclei, the eggs were incubated with Hoechst 33258 overnight at 4°C and washed with PBS plus 0.1% Triton X-100.

### Sequence alignment

Protein sequences for human TMEM132 family of proteins and *Drosophila melanogaster* dtn were downloaded from http://www.ensembl.org. Pair-wise comparisons were made with BLAST. Multi-sequence alignment was performed with DNAStar Lasergene using ClustalW method.

### Fly image acquisition

The images of the flies and embryos were acquired on a Zeiss Discovery microscope using a QImaging micropublisher camera. The nuclei staining with Hoechst 33258 was visualized with a Nikon E600 fluorescence microscope.

Strains and plasmids are available upon request. The authors affirm that all data necessary for confirming the conclusions of the article are present within the article, figures, and tables.

## Results and discussion

### *Drosophila dtn* protein share conserved sequence and structural homology with mammalian TMEM132 family proteins

**Fig 1:**
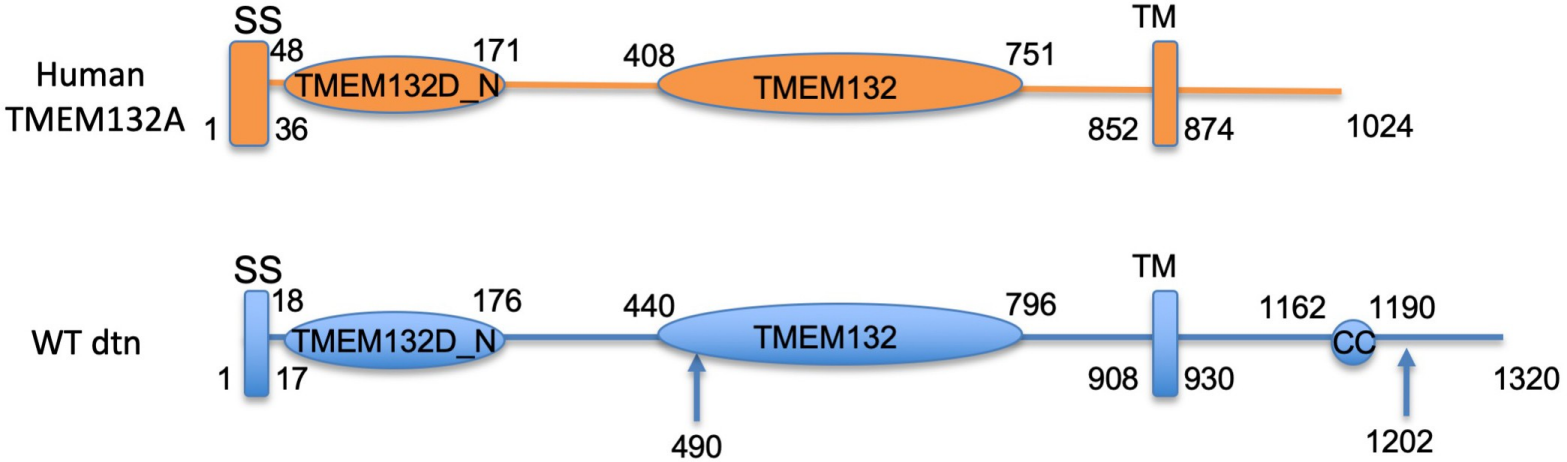
Domain structure of the human TMEM132A and *Drosophila* dtn protein. Both proteins contain an N-terminal signal peptide sequence (SS), a conserved extracellular TMEM132D_N and a TMEM132 domain, a transmembrane (TM) domain. The *Drosophila* dtn protein contains an intracellular coiled-coil (CC) domain that is missing in human TMEM132 proteins. The structure prediction was made using the SMART (http://smart.embl-heidelberg.de/) protein sequence analysis tool. Arrows point to the positions on the peptide where mutations were generated via CRISPR/Cas9 in this work.

To design a strategy to mutate *Drosophila dtn* gene, we first compared the peptide sequence of dtn to those of human TMEM132 proteins. SMART (http://smart.embl-heidelberg.de/) protein structure analysis suggested that this 1320-amino-acid protein contained a N-terminal signal peptide, two extracellular motifs conserved among TMEM132 proteins (TMEM132D_N and TMEM132 domains), a transmembrane domain (TM), and a coiled-coil domain in the intracellular tail (Fig 1). Pairwise and multisequence alignment indicated that dtn shared sequence homology with human TMEM132 families in the extracellular and transmembrane domains (~25–27% identity and ~40–43% similarity; Figs 1 and S1 and Table 3). However, the intracellular domains of human TMEM132 proteins were much shorter than that of dtn, and did not carry the coiled-coil domain, suggesting that there may be different downstream pathways in the two species.

**Table 3:** dtn share homology with human TMEM132 proteins.

		TMEM132A isoform a (22–914)	TMEM132B isoform a (42–949)	TMEM132C isoform X1 (31–955)	TMEM132D precursor (49–971)	TMEM132E precursor (55–917)
dtn isoform b (5–988)	identity	25%	26%	27%	26%	27%
	similarity	40%	43%	43%	43%	42%

### Efficient deletion of multiple exons of dtn through a polycistronic transcript-based multiplex CRISPR/Cas9 approach

**Fig 2:**
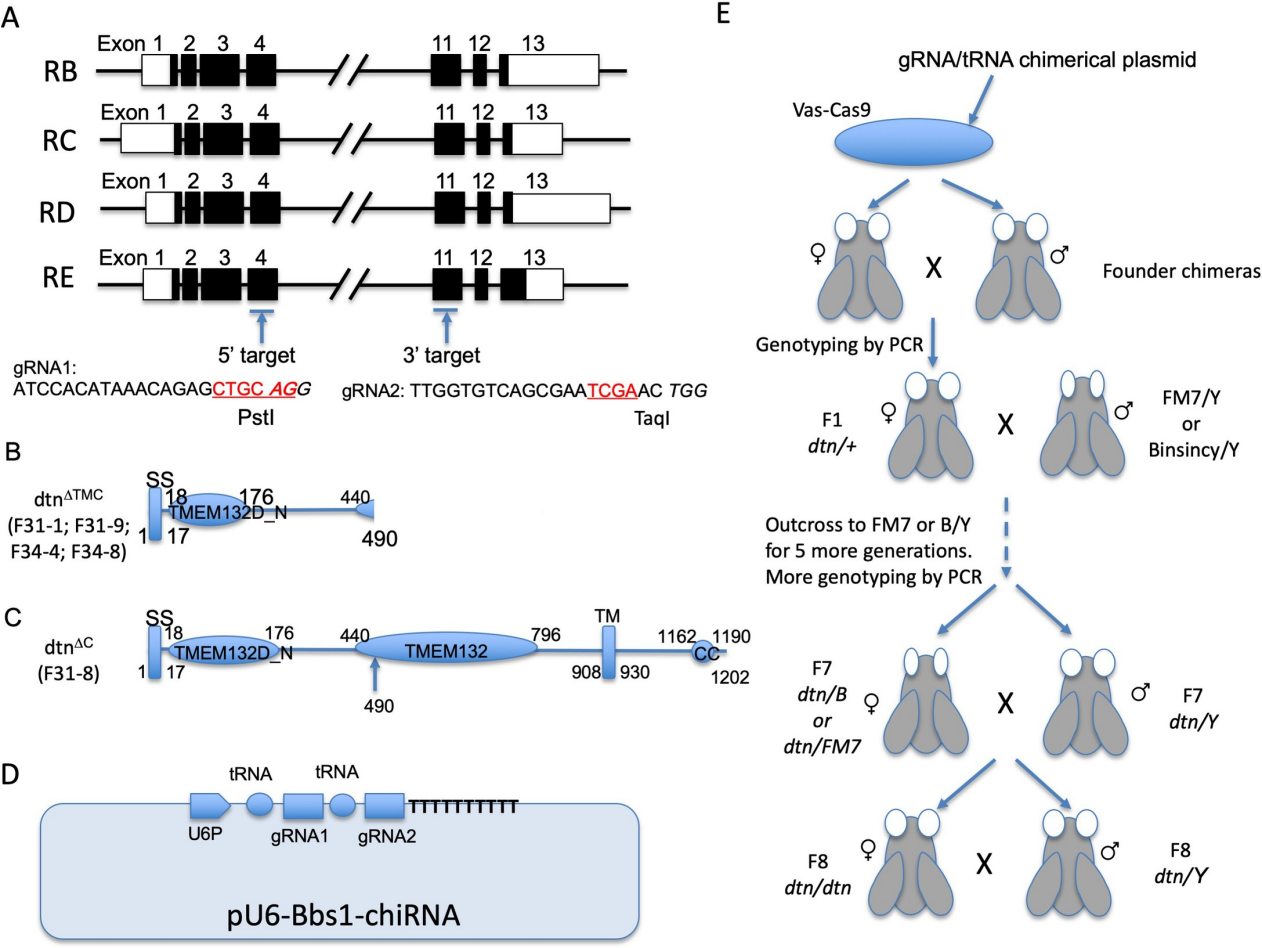
Mutagenesis strategy and screening scheme for generating multiple *dtn* alleles. (A) Genomic structure of the four splice variants of *Drosophila* dtn based on http://ensembl.org. Two gRNAs were selected to target exon 4 (gRNA1) and exon 11 (gRNA2) shared among all splice variants of *Drosophila dtn*. Open boxes denote untranslated regions. Solid boxes denote the coding region. The target sites for PstI and TaqI are in red letters. (B) Only the first 490 residues of the protein, including SS, TMEM132D_N and part of the TMEM132 domains, are present in mutant alleles F31-1, F31-9, F34-4 and F34-8, due to the removal of the TM and intracellular domains (dtn^ΔTMC^). (C) Two residues at position 490 and 119 residues at the C-terminus of dtn are removed in F31-8 (dtn^ΔC^). (D) The two gRNAs (gRNA1 and gRNA2) separated by tRNAs were cloned into pU6-Bbs1-chiRNA such that a single polycistronic tRNA-gRNA chimerical transcript will be made using a U6 promoter (U6P). (E) The mutagenesis/screening/breeding scheme. *Vas-Cas9* transgenic flies injected with the *dtn* gRNA/tRNA chimerical plasmid were crossed to siblings, and were euthanized and genotyped by PCR after pupae were seen in the vial. F1 females were then PCR genotyped and outcrossed to balancer males for five generations to establish stable mutant lines, after which hemizygous mutant male and homozygous female flies were obtained in subsequent generations.

Based on the published genome annotation (http://ensembl.org), there are 13 exons in the *Drosophila dtn* gene. Four transcript variants (RB, RC, RD and RE) were predicted with alternative use of exons and variable lengths of the untranslated regions (Fig 2A). We decided to simultaneously target two sites shared by all four variants of the gene, in exon 4 and exon 11, respectively (Fig 2A). Ideally, simultaneous targeting of both sites would lead to a deletion spanning from amino acid residue 490 to 1202 of the dtn protein, including the TM region (Fig 2B). A frameshift resulting from the deletion may allow the deletion of the remaining C-terminal region of the protein. Additional alleles could be produced through NHEJ in exon 4, leading to similar removal of the TM and intracellular regions as the result of a frameshift. These were likely null alleles given the removal of the TM domain, the entire intracellular domain, and nearly half of the extracellular domain. Theoretically, it could be possible that the truncated protein may exhibit dominant negative activities, but our phenotypical analysis (see below) did not reveal any dominant phenotype. Alternatively, short deletions in exon 11 may lead to the removal of the last ~100 intracellular residues, which might impact the function of the protein dependent on how important these residues are (Fig 2C).

Using the online target selection tool CRISPOR (http://crispor.tefor.net/), we selected two targets with high specificity scores and few potential off-targets. To facilitate screening and subsequent genotyping, the two targets we selected also contained restriction sites proximal to the PAM (protospacer adjacent motif) sequence. For exon 4, we chose ATCCACATAAACAGAGCTGC *AGG*, which contains a PstI site. For exon 11, we chose TTGGTGTCAGCGAATCGAAC *TGG*, which contains a TaqI site.

To ensure efficient mutagenesis at both targets, we decided to use the multiplex CRISPR method newly developed by Xie et al [22]. In this method, a chimerical transcript comprising alternating tRNAs and gRNAs was produced from a shared promoter. Subsequent processing of the tRNAs by RNaseD and RNaseP released gRNAs, allowing simultaneous mutagenesis of multiple targets. We assembled a plasmid that expressed from a U6 promoter a chimerical RNA comprising the two gRNAs targeting exon 4 and exon 11 of *dtn*, separated by tRNAs (Fig 2D; see materials and methods for details of the plasmid assembly).

**Fig 3:**
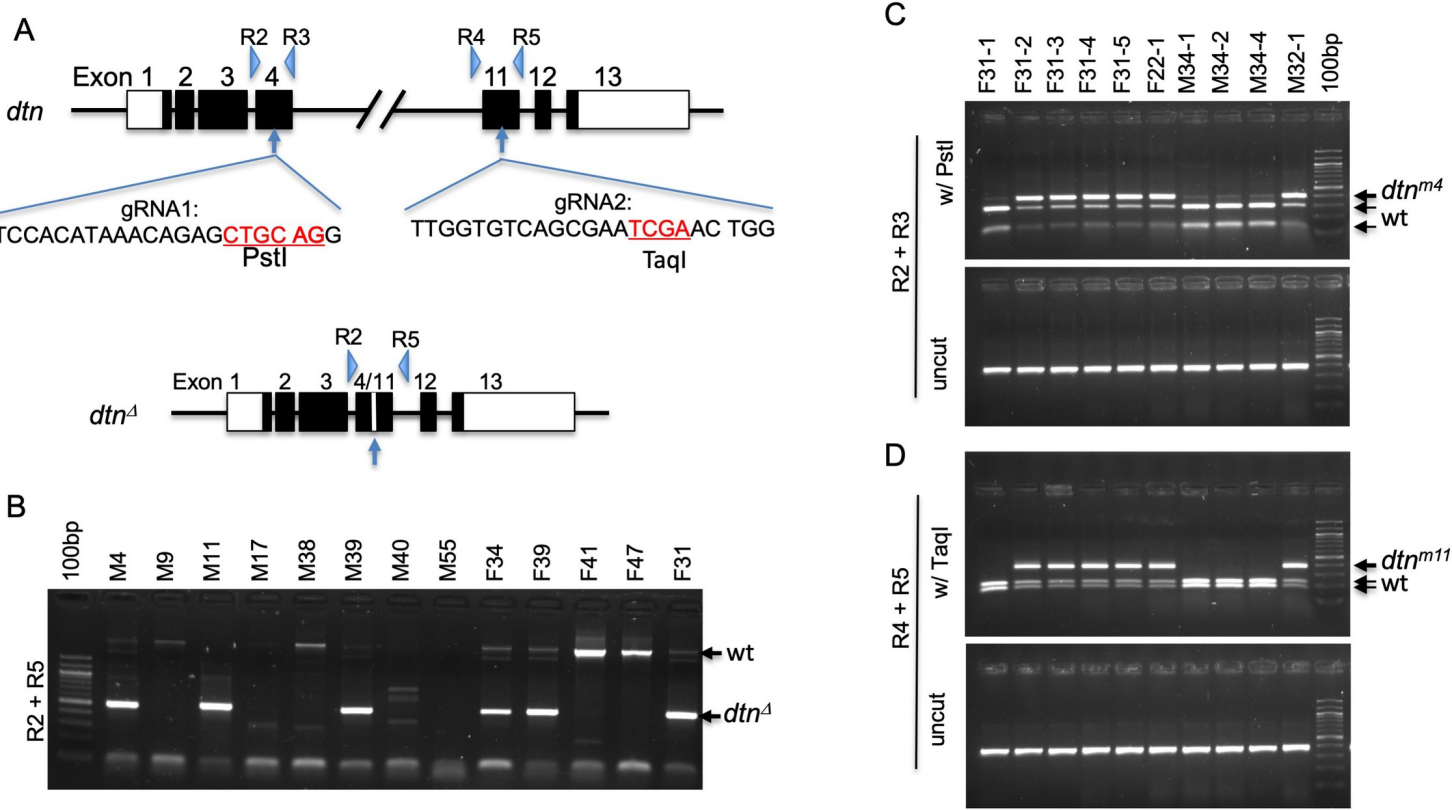
Strategy for screening *dtn* mutant alleles. (A) Two pair of primers were designed to amply the genomic regions surrounding the gRNA1 (R2 and R3) and gRNA2 (R4 and R5) targets. Mutations in these target sites would disrupt the PstI and TaqI sites, respectively. PCR with R2 and R5 would amplify a longer product in wild type, and a shorter product after the genomic region between the two gRNA targets was removed (*dtn*^*Δ*^). Open boxes denote untranslated regions. Solid boxes denote the coding region. The target sites for PstI and TaqI are in red letters. (B) PCR with the R2/R5 primer combination allowed the identification of the deletion of DNA between the two gRNA targets. Each lane contained PCR product with genomic DNA extracted from a single founder fly as template. wt: 3045bps; *dtn*^*Δ*^: 503bps. (C) PCR with the R2/R3 primer combination followed with PstI digestion allows the identification of indels at the gRNA1 target in exon 4. Each lane contained PCR product genomic DNA extracted from a single F1 female fly as template. wt: 287bps and 106bps; *dtn*^*m4*^: 394bps. (D) PCR with the R4/R5 primer combination followed with TaqI digestion allows the identification of indels at the gRNA2 target in exon 11. Each lane contained PCR product genomic DNA extracted from a single F1 female fly as template. wt: 216bps and 165bps; *dtn*^*m11*^: 382bps.

To generate mutant alleles of *dtn* in the *Drosophila* germline, we had the tRNA-gRNA chimerical plasmid injected into a *Drosophila* stock expressing Cas9 in the germline (Fig 2E). Among the ~ 300 eggs injected, ~120 developed into larvae. Among these, 55 male and 42 female founders were set up for mating (total 97), and 6 dead founders were collected for genotyping (grand total = 103). We crossed each virgin founder female to one founder male (Fig 2E). For non-virgin female founders, we housed them individually with males carrying FM7 or Binsinscy balancer chromosomes. For extra founder males, we crossed them with virgin females carrying a FM7 or Binsinscy balancer and a lethal deficiency. When wandering larvae and/or pupae appeared in the vials, we sacrificed the founders and screened for deletion of the region between exon 4 and exon 11 of *dtn* with PCR using primers R2 and R5 (Fig 3A and Table 2).

Among the 97 live founders, 56 were fertile. Through PCR using primers R2 and R5, a product of ~500 bps was amplified in 21 (out of 56 fertile) founders, suggesting expected deletion of the exons between the two CRISPR targets (Fig 3A and 3B). PCR products that were shorter or longer than expected were amplified in 11 (out of 56 fertile) additional founders (e.g., M17, M38 and M40; Fig 3B), likely reflecting deletions/duplications at the two CRISPR targets in addition to the deletion of the region between them. These results indicated that the efficiency of the successful deletion between the two targets was at 37.5–57.1%, suggesting that mutagenesis at multiple sites can be achieved *in vivo* in *Drosophila* with high efficiency using a polycistronic tRNA-gRNA-based approach.

### More mutant alleles produced through non-homologous end joining at both CRISPR targets

Because the founder flies had chimerical germlines comprising a variety of gametes harboring unique mutations in *dtn*, another round of screening was needed to establish stable fly strains carrying the desired mutation. We collected virgin F1 female progeny of the founders from which deletion of the *dtn* exons were detected from the primary screen, and bred them with FM7- or Binsinscy-carrying males. Among 39 F1 females, 4 had the expected deletion between exon 4 and exon 11 (Table 4).

**Table 4:** F1 screen revealed high indel and deletion efficiency.

Parents (F x M)	Total F1 vial set up	Deletion Carriers	5’ target indel	3’ target indel
F31 x M1	16	3/16	6/6	5/6
F22 x FM7	1	0/1	1/1	1/1
M32 x B	8	0/8	6/7	4/7
M39 x B	1	0/1	1/1	0/1
F34 x M4	10	1/10	1/3	1/3
F32 x M2	1	0/1	---	---
M37 x B	2	0/2	---	---
Total	39	4/39	15/18	11/18

**Fig 4:**
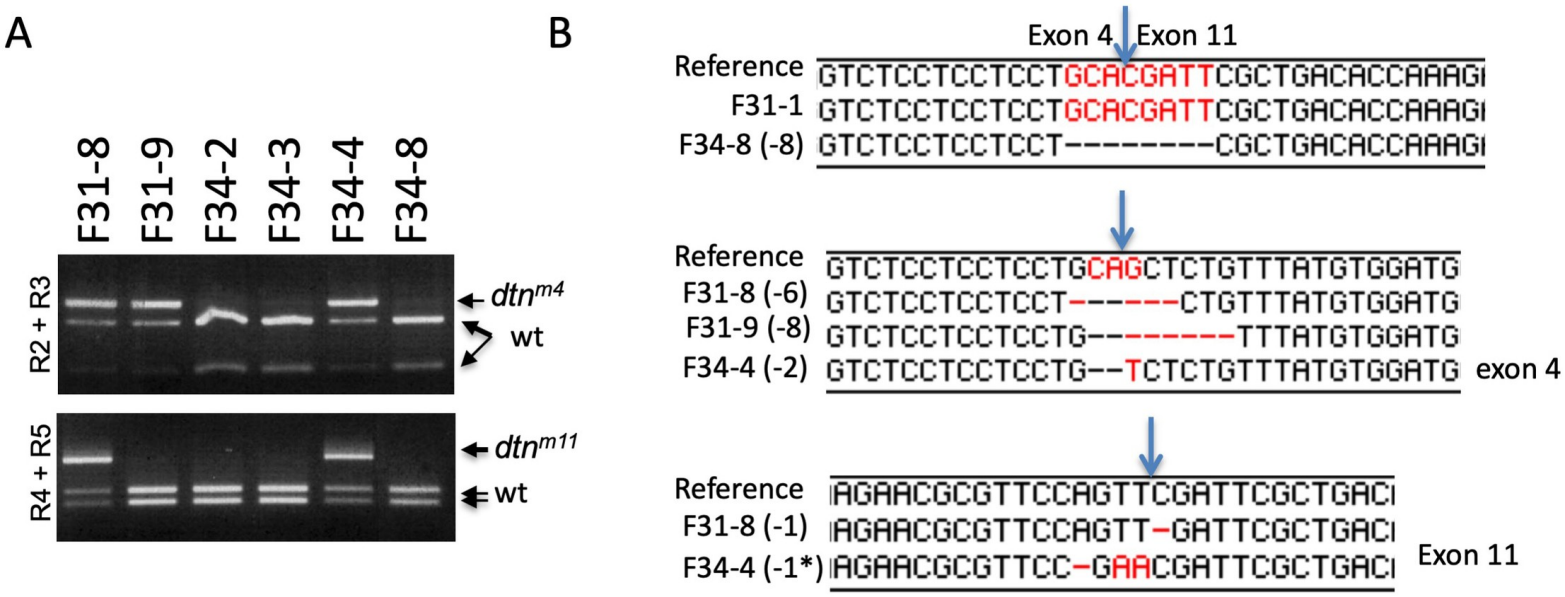
Sequencing analysis of five mutant *dtn* alleles. (A) Genotyping results for F1 offspring derived from F31 and F34 founder flies. For F31-8, F31-9 and F34-4, the top band (PstI resistant) was purified and sequenced. For F31-8 and F34-4, the top band (TaqI resistant) was purified and sequenced. For F31-1 and F34-8, the PCR products with R2/R5 primer combination were purified and sequenced. (B) Sequence of the mutated *dtn* locus in F31-1, F34-8 (multi-exon deletions), F31-8, F31-9 and F34-4 (indels in exon 4 and/or exon 11). The reference genomic sequences were from http://ensembl.org. The alignments were done in Lasergene DNAStar. Arrows point to predicted Cas9 cut sites.

To obtain additional mutant alleles for *dtn*, and information on the mutagenesis efficiency at each CRISPR target, we amplified the genomic regions surrounding the targets in exon 4 (using primers R2 and R3) and exon 11 (using primers R4 and R5), respectively (Figs 3A, 3C and 4A). PstI cut of the exon 4 PCR product and TaqI cut of the exon 11 product resulted in two shorter DNA fragments in wild type (Figs 3C and 4A). In 15 out of 18 F1 females, half of the exon 4 PCR products were resistant to PstI, suggesting successful mutations disrupting the PstI site (Figs 3C and 4A and Table 4). Similarly, 11 out of 18 F1 females carried mutations to the TaqI site in exon 11 (Figs 3C and 4A and Table 4).

To more accurately specify the mutations in the *dtn* mutant flies, we sequenced the PCR products from five F1 females. As indicated in Fig 4B, the CRISPR targets in exon 4 and 11 were cut by Cas9 and the ends were joined without further mutation in F31-1. In contrast, 8 bps were removed before the ends were joined in F34-8. F31-9 and F34-4 carried short deletions in exon 4 (8 bps in F31-9, 2 bps in F34-4) resulting in frame shifts. On the other hand, 6 bps were removed in exon 4 of *dtn* in F31-8, thus leaving the reading frame intact with 2 amino acids removed. In addition, F31-8 and F34-4 also carry frame-shifting small deletions in exon 11 (1 bp removed in both, and two Ts were converted to As in F34-4). Therefore, we predict that we have obtained 4 alleles for *dtn* (F31-1, F31-9, F34-4 and F34-8) that are missing the TM domain and intracellular domain, as well as about half of the extracellular domain (Fig 2B). F31-8 had 2 residues in the extracellular domain and the last ~100 residues at the C terminus removed.

### No off-target mutations were found in dtn mutant flies

A major concern associated with CRISPR/Cas9-based genome engineering is the contribution of potential off-target mutations to the phenotype observed in the mutant animals. To ease this concern, we first examined whether the two gRNAs we used to generate *dtn* mutants caused off-target mutations. One website (http://targetfinder.flycrispr.neuro.brown.edu/index.php) predicted three potential off-targets for the gRNA targeting exon 4 of *dtn*, and one potential off-target for the gRNA targeting exon 11 of *dtn* (Table 5). Three of these potential off-targets were in intergenic regions and the fourth one was in a large intron (Table 5), thus mutations at these sites were unlikely to affect any gene function. Nevertheless, we sequenced all four potential off-targets in F34-8 homozygous females and found no mutations in the potential off-targets and surrounding sequences, suggesting that the phenotypes observed in the *dtn* mutant flies were the results of loss of *dtn* function rather than off-target mutations.

**Table 5:** No off-target mutations found in Line F34-8.

Targets	Sequence	location	context	Results
On-target #1	ATCCACAT|AAACAGAGCTGC AGG	X:6227987‥6228009	dtn exon 4	
off-target #1	AggtcCAg|AAACAGAGCTGC CGG (5 mismatches)	3L:19339801‥19339823	intergenic	No mutation found
off-target #2	gaggtggT|AAACAGAGCTGC GGG (7 mismatches)	2L:5699229‥5699251	intergenic	No mutation found
off-target #3	Aaattgga|AAACAGAGCTGC TGG (7 mismatches)	2R:8820617‥8820639	sns, intron 3	No mutation found
On-target #2	TTGGTGTCAGCGAATCGAAC *TGG*	X:6230573‥6230595	dtn exon 11	
off-target #4	caaactgg|AGCGAATCGAAC TGG (8 mismatches)	3L:6831508‥6831530	intergenic	No mutation found!

To further avoid the impact of possible lesions in other parts of the genome caused by our mutagenesis manipulation, we outcrossed one of the *dtn* mutant lines, F34-8, to FM7/Y males for six generations (Fig 2E). No significant changes in phenotype were observed after outcrossing, indicating that the phenotype observed was indeed the result of loss of dtn function.

### Both maternal and paternal dtn contribute to *Drosophila* development

**Fig 5:**
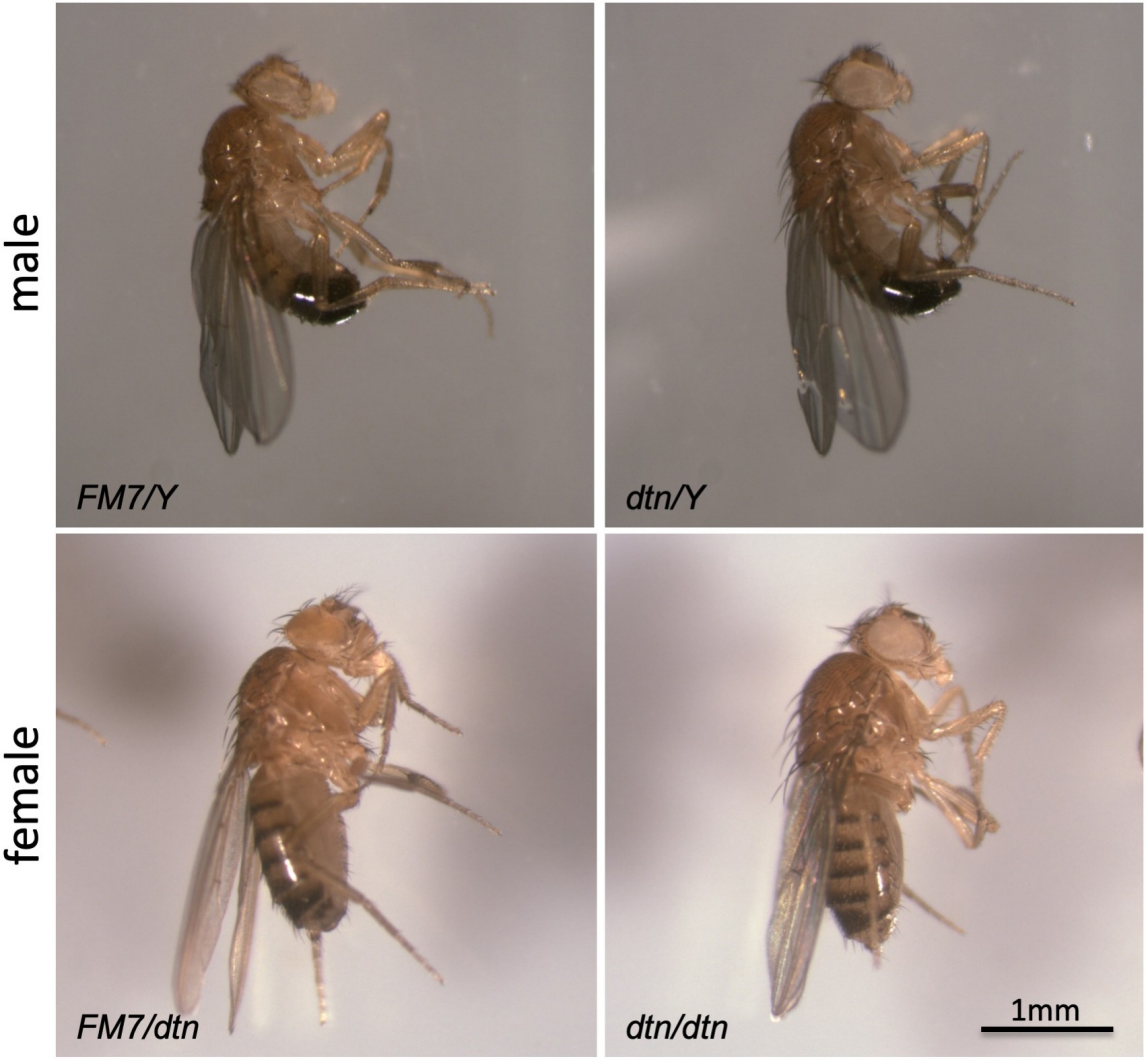
Normal morphology of *dtn* mutant flies. Lateral views of adult flies are shown. Five day old adult flies from the F34-8 line were anaesthetized and euthanized, brought to room temperature for 30 minutes, and visualized with a Zeiss Discovery microscope.

To determine the requirement for *dtn* in *Drosophila* development, we examined the progeny of the heterozygous *dtn* mutant female flies and males carrying the X balancers (Fig 2E). If zygotic *dtn* was essential for survival, we expected to see no or reduced number of male flies carrying the *dtn* mutations. Alternatively, we expected to observe abnormal morphology in mutant male flies, if *dtn* was required for morphogenesis. As shown in Table 6 and Fig 5, flies carrying *dtn* mutations represented roughly half of the male progeny (e.g., *dtn/Y* flies represented 55.5% of progeny for F31-1 and 51.1% for F34-8), and they did not exhibit any abnormal morphology. This suggested that zygotic *dtn* was not essential for the survival and morphogenesis of male flies.

**Table 6:** Progeny derived from the crosses between *dtn/B (dtn/FM7)* females and B/Y (or FM7/Y) males.

Type of alleles	allele name	female			male		
		*B/B (FM7/FM7)*	*dtn/B (dtn/FM7)*	total	*B/Y (FM7/Y)*	*dtn/Y*	total
Deletion	F31-1	104	145	249	53	66	119
	F34-8	59	121	180	69	72	141
5’ frameshift	F31-9	11	15	26	17	17	34
	F34-4	15	60	75	30	44	74
3’ frameshift	F31-8	28	37	65	25	17	42

To further determine the requirement for *dtn* in male fertility and female development, we crossed the heterozygous female carriers with mutant male flies (Fig 2E). As shown in Table 7, mutant males of all five *dtn* alleles were fertile, and both *dtn/dtn* females and *dtn/Y* males were present in progeny. However, the number of *dtn/dtn* females was lower than that of *dtn/FM7* females (e.g., *dtn/dtn* flies represent 24.3% of female progeny for F31-1). This appeared to indicate that *dtn/dtn* females were less viable than control females.

**Table 7:** Progeny derived from the crosses between *dtn/FM7* females and *dtn/Y* males.

Type of alleles	allele name	female			male		
		*dtn/B (or dtn/FM7)*	*dtn/dtn*	total	*B/Y (or FM7/Y)*	*dtn/Y*	total
Deletion	F31-1	106	34	140	45	42	87
	F34-8	66	38	104	25	31	56
5’ Frameshift	F31-9	62	39	101	47	32	79
	F34-4	38	8	46	16	15	31
3’ Frameshift	F31-8	50	19	69	20	24	44

Next, we sought to determine whether removing both maternal and paternal *dtn* would affect *Drosophila* development. To do this, we crossed *dtn/dtn* mutant female and *dtn/Y* male flies from F34-8 (Fig 2E). Among the 10 breeding vials we set up (2–3 males with 3–5 females each), normal-looking flies (both males and females) were hatched in 4 vials, but not in the other 6 vials, suggesting that loss of both maternal and paternal *dtn* lead to a partially penetrant reproductive defect.

**Fig 6:**
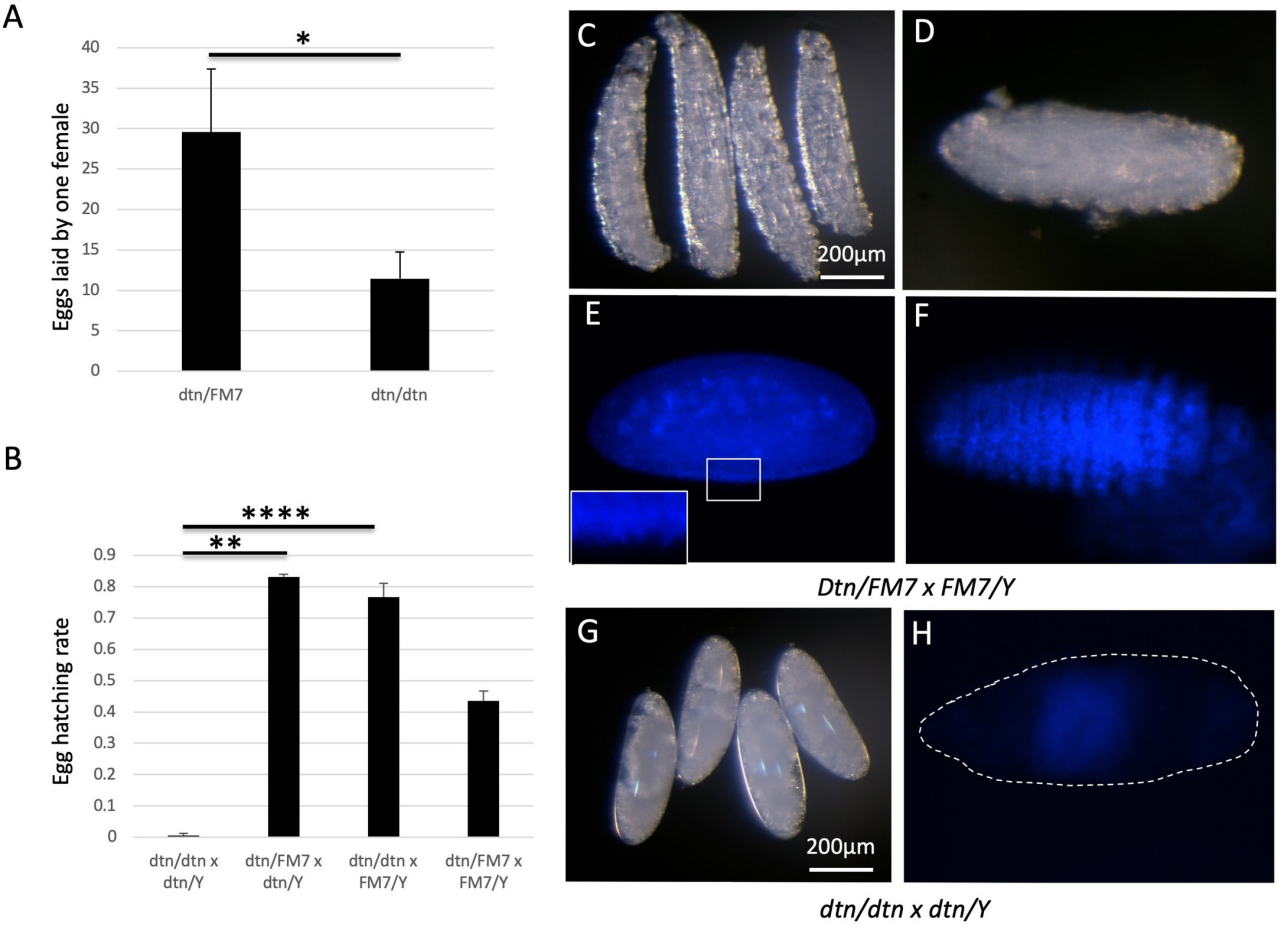
Both maternal and zygotic dtn contributed to embryonic development. (A) *dtn/dtn* female flies laid fewer eggs than *dtn/FM7* flies. (B) Few eggs from the cross between *dtn/dtn* female and *dtn/Y* hatched while most eggs from the control crosses hatched. See Materials and Methods for the egg-laying experiment procedure. For both A and B, pair-wise comparisons were made with two-tailed unpaired student t-test. *: p<0.05, **: p <0.01, ****: p <0.0001. (C, D) After 24 hr. incubation at 25°C, many larvae were present in control crosses (C). Some unhatched embryos were clearly segmented (D). (E) A blastoderm stage embryo showing nuclei on its surface. The inset showed the close up view of the rectangular region. (F) DNA staining shows the segmented embryo. (G) Eggs from the cross between *dtn/dtn* females and *dtn/Y* males did not show segmentation. (H) No individual nucleus was visible in eggs from the cross between *dtn/dtn* females and *dtn/Y* males, suggesting that they arrest before the blastoderm stage. C, D and G showed the darkfield images of dechorionated larvae/embryos. E, F and H showed the nuclei in the embryos stained with the DNA dye Hoechst 33258.

The scarcity of offspring from the cross between *dtn/dtn* females and *dtn/Y* males could be due to defects in oogenesis in *dtn/dtn* females, resulting in fewer eggs. To test whether this is the case, we set up breeding vials containing 10~15 females and 3~5 males. After feeding them with extra yeast for 3 days, we allowed them to lay eggs on apple juice plates, and counted the eggs and larvae after 24 hours. We found that each *dtn/FM7* female laid on average 29.5 eggs within a 24-hour period, whereas each *dtn/dtn* female laid on average 11.4 eggs within the same time (Fig 6A). This suggested that *dtn/dtn* mutant females did lay significantly fewer eggs than heterozygotes (p = 0.033, student t-test).

To investigate whether the eggs laid by *dtn/dtn* females hatched at the same rate as those laid by *dtn/FM7* females, we incubated the eggs for 48 hours and counted the number of larvae and eggs afterwards. We found that over 80% of the eggs laid by *dtn/FM7* females crossed to *dtn/Y* males hatched, whereas only 0.5% of the eggs laid by *dtn/dtn* females crossed to *dtn/Y* males hatched (Fig 6B). Interestingly, we found that 77% of the eggs laid by *dtn/dtn* females crossed to *FM7/Y* males hatched, suggesting that zygotic dtn alone was sufficient to support embryonic development.

To further reveal when the development of *dtn/dtn* mutant embryos was arrested, we inspected the embryos. Dark field images revealed the morphology of larvae (Fig 6C) and segmented embryos (Fig 6D) in control cross between *dtn/FM7* and *FM7/Y* flies. With the help of DNA dye Hoechst 33258, we were able to visualize blastoderm stage (Fig 6E), as well as segmented embryos (Fig 6F). The eggs from the cross between *dtn/dtn* female and *dtn/Y* male flies did not show segments (Fig 6G). Hoechst 33258 staining only showed diffuse weak signal in the center of the egg (Fig 6H), suggesting that these eggs were arrested prior to the blastoderm stage, when nuclear staining on the surface of the normal embryo was clearly visible (see Fig 6E).

### Deficiencies over the dtn locus failed to complement for the loss of dtn in *Drosophila* reproduction

**Fig 7:**
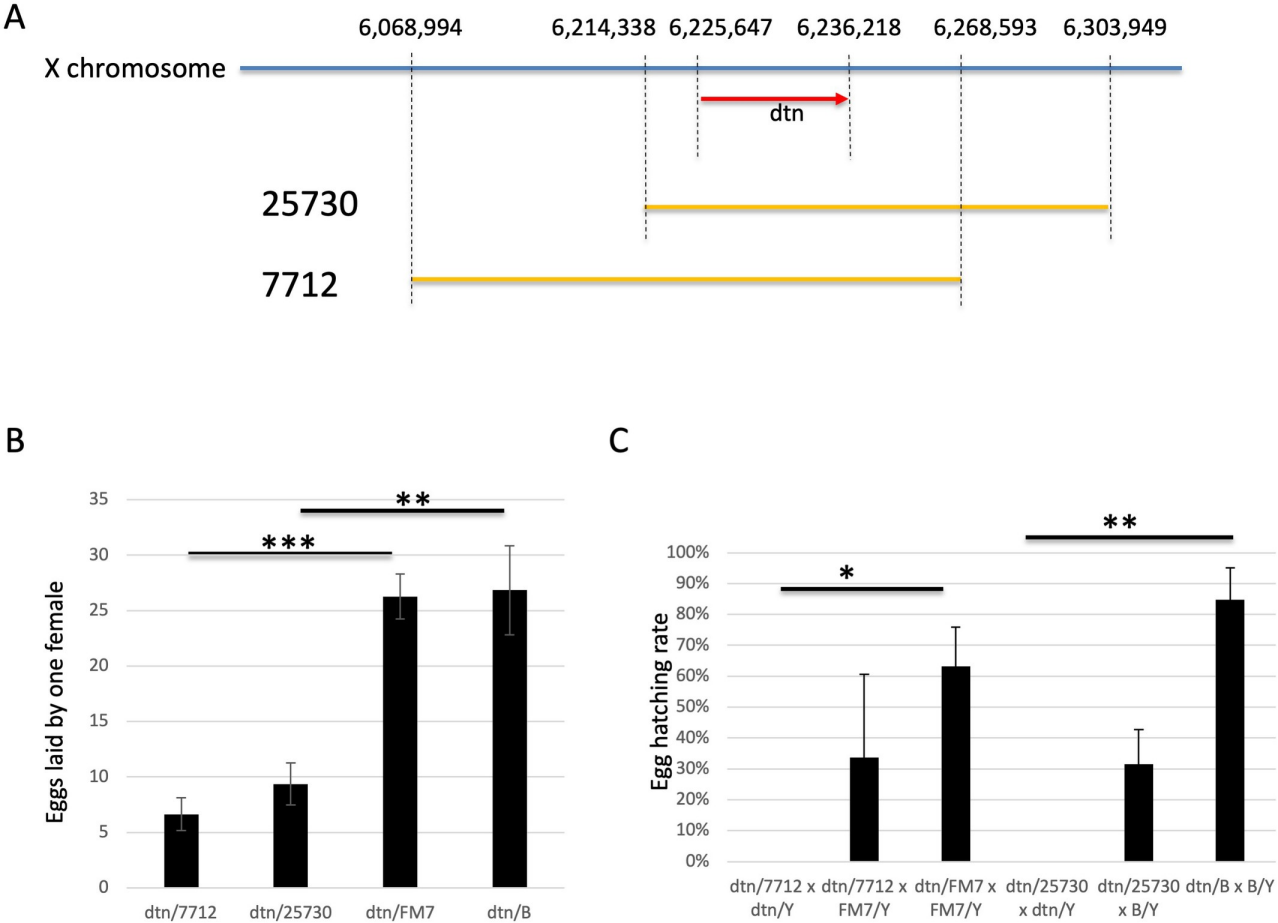
Deficiencies over the *dtn* locus failed to complement for the loss of *dtn* in *Drosophila* reproduction. (A) A schematic illustration (not drawn to scale) showing the *dtn* locus (red arrow showing the orientation of the transcription) and the two deficiencies used for the complementation tests (orange lines). Numbers indicate the locations of the boundaries of the deficiencies and the *dtn* locus on the X-chromosome (blue line). (B) *dtn/7712* and *dtn/25730* female flies laid fewer eggs than *dtn/FM7* and *dtn/B* flies, respectively. (C) No egg from the cross between *dtn/7712* (or *dtn/25730*) females and *dtn/Y* males hatched while most eggs from the control crosses hatched. See Materials and Methods for the egg-laying experiment procedure. For both B and C, pair-wise comparisons were made with two-tailed unpaired student t-test. *: p<0.05, **: p<0.01, ***: p <0.001.

To further rule out the possibility of an off-target mutation, rather than the loss of dtn function per se, led to the observed defects in reproduction, we performed complementation tests using *Drosophila* strains with known deficiencies over the *dtn* locus. As indicated in Fig 7A, *Drosophila dtn* locus spans from 6,225,647 to 6,236,218 of X chromosome. Line 7712 from the Bloomington Drosophila Stock Center carries a deficiency spanning from 6,068,994 to 6,268,593 of X chromosome. Another deficiency line, 25730, carries a deficiency spanning from 6,214,338 to 6,303,949 of X chromosome (data from http://bdsc.indiana.edu). Both deficiencies include the *dtn* locus, and should not complement the reproduction defects exhibited by the *dtn* mutants, unless those defects were linked to another part of the genome as an off-target effect of the mutagenesis treatment.

We first bred *7712/FM7* and *25730/B* virgin females to *dtn/Y* males. Interestingly, much fewer *dtn/7712* (n = 62) females than control *dtn/FM7* females (n = 120) were recovered from the same cross, suggesting reduced viability due to the loss of *dtn* (Table 8). Similarly, fewer *dtn/25730* (n = 44) females than control *dtn/B* females (n = 102) were recovered, further supporting an important role of *dtn* in fly viability (Table 8).

**Table 8:** Progeny derived from the crosses between deficiency (*25730/B* or *7712/FM7*) females and *dtn/Y* males.

Parents		female			male		
Female	male	*dtn/FM7 (or dtn/B)*	*Dtn/7712 (or dtn/25730)*	total	*B/Y (or FM7/Y)*	*Df/Y*	total
*7712/FM7*	*dtn/Y*	120	62	182	66	0	66
*25730/B*	*dtn/Y*	102	44	146	63	2	65

Next, we crossed *dtn/7712 (*or *dtn/25730)* and control *dtn/FM7 (*or *dtn/B)* virgin females with *dtn/Y* or *FM7/Y* (or *B/Y*) males, and counted the eggs and larvae. We found that *dtn/7712* females laid fewer eggs (6.64 eggs per female per 24 hrs) than control *dtn/FM7* females (26.27 eggs per female per 24 hrs) (Fig 7B, p = 0.00016, student t-test). Similarly, *dtn/25730* females also laid fewer eggs (9.36 eggs per female per 24 hrs) than control *dtn/B* females (26.86 eggs per female per 24 hrs) (Fig 7B, p = 0.0015, student t-test). These observations are consistent with the observation that *dtn/dtn* females laid fewer eggs than *dtn/FM7* females (see Fig 6A), suggesting that the oogenesis defects result from the loss of dtn function, not an off-target effect.

Finally, we incubated the eggs at 25°C for 48 hrs to see how many hatched. We found that majority of the eggs laid by *dtn/FM7* (63%) or *dtn/B* (85%) females hatched in 48 hrs (Fig 7C). In contrast, when *dtn/7712* or *dtn/25730* females were crossed to *dtn/Y* males, no egg hatched, strongly supporting the conclusion that both maternal and zygotic *dtn* is important for development beyond the embryonic stage (Fig 7C, p = 0.019 and 0.0075, respectively, student t-test). We also observed reduced hatching rate for the cross between *dtn/7712* and *FM7/Y* (34%) or the one between *dtn/25730* and *B/Y* (32%) compared to the control crosses between *dtn/FM7* (or *dtn/B*) and *FM7/Y* (or *B/Y*), although the difference failed to reach statistical significance (p = 0.22 and 0.058, respectively). This reduction in hatching rate may partly result from failed development of most *7712/Y* and *25730/Y* embryos.

In this study, we show that deletions between two targets ~3 kb apart can be achieved at an efficiency of 37–57% using the polycistronic tRNA-gRNA approach. Mutagenesis efficiency at an individual site is on average above 65%, and in the best cases (such as in F31) can reach nearly 100%. These results suggest that this approach is ideal in simultaneously producing multiple alleles, including relatively large deletions, of a gene of interest.

TMEM132 family of transmembrane proteins have been associated with a variety of neurological, respiratory and circulatory disorders, as well as embryonic development and cancer [6–9, 11, 13, 14, 23, 24], thus understanding the function of these proteins is of great value to human health. Unfortunately, the presence of five highly homologous *Tmem132* genes in the mouse and human genomes suggests potential functional redundancy between paralogues, making it challenging to reveal the full requirement for these proteins in vivo. *Dtn* is the only TMEM132 homolog in the *Drosophila* genome, allowing us to investigate its function in vivo.

Our data suggest an important role for maternal *dtn* in oogenesis as *dtn/dtn* mutant (as well as *dtn* over deficiencies) female flies laid significantly fewer eggs than *dtn/FM7* heterozygotes. However, in the presence of *FM7/Y* males, most of the eggs laid by *dtn/dtn* mutant flies hatched into larvae and eventually adult flies, suggesting that maternal *dtn* is not required for embryonic development. Interestingly, eggs lacking both maternal and paternal dtn failed to reach the blastoderm stage, suggesting that dtn is essential for *Drosophila* reproduction. It is possible that the eggs laid by *dtn/dtn* mutants were not fertilized and/or activated by sperms of the *dtn/Y* males. If so, this could reflect a behavioral defect preventing successful mating, and could correspond to a previous report on *dtn* expression in the nervous system [15, 25]. Alternatively, there may be defects in gamete interaction or intracellular signaling leading to egg activation. Consistent with this possibility, the dtn protein was detected in mature oocyte and appeared to be phosphorylated upon egg activation [26]. Alternatively, zygotic transcription of wild type *dtn* provided by FM7/Y male might allow the development of the eggs laid by *dtn/dtn* female flies. However, this scenario is less likely given the eggs lacking maternal and zygotic dtn failed to reach blastoderm stage, when large-scale zygotic transcription occurs. Further investigation is needed to explore the roles of dtn in the early development of *Drosophila* embryos.

## Supporting information

S1 Fig Alignment of peptide sequences of *Drosophila* dtn and the human TMEM132 family members. All peptide sequences were downloaded from http://ensembl.org. The multiple alignment was performed using Lasergene DNAStar with the Clustal W method. Dash lines in individual protein sequence indicate gaps, whereas dash lines in the Majority sequence indicate lack of consensus residue at corresponding position.
(ZIP)

S2 Fig Whole gel photos. (A) Whole gel photo for Fig 2B. (B) Whole gel photo for Fig 2C. (C) Whole gel photo for Fig 2D. (D) Scanned gel image from the original notebook. The digital file for the whole gel photo was not saved.
(TIF)
