# Correction: Efficient multiplexed genome engineering with a polycistronic tRNA and CRISPR guide-RNA reveals an important role of detonator in reproduction of *Drosophila melanogaster*

**DOI:** 10.1371/journal.pone.0250188

**Published:** 2021-04-08

**Authors:** Cristin Chon, Grace Chon, Yurika Matsui, Huiqing Zeng, Zhi-Chun Lai, Aimin Liu

The following information is missing from the Funding statement: This study was supported by the NIH in the form of a grant awarded to AL (R03-HD101765).
