# Supplementary figures and images for: Efficient multiplexed genome engineering with a polycistronic tRNA and CRISPR guide-RNA reveals an important role of detonator in reproduction of Drosophila melanogaster
Source: PLoS One. 2021 Jan 14;16(1):e0245454. doi: 10.1371/journal.pone.0245454 (PMC7808601; doi:10.1371/journal.pone.0245454)

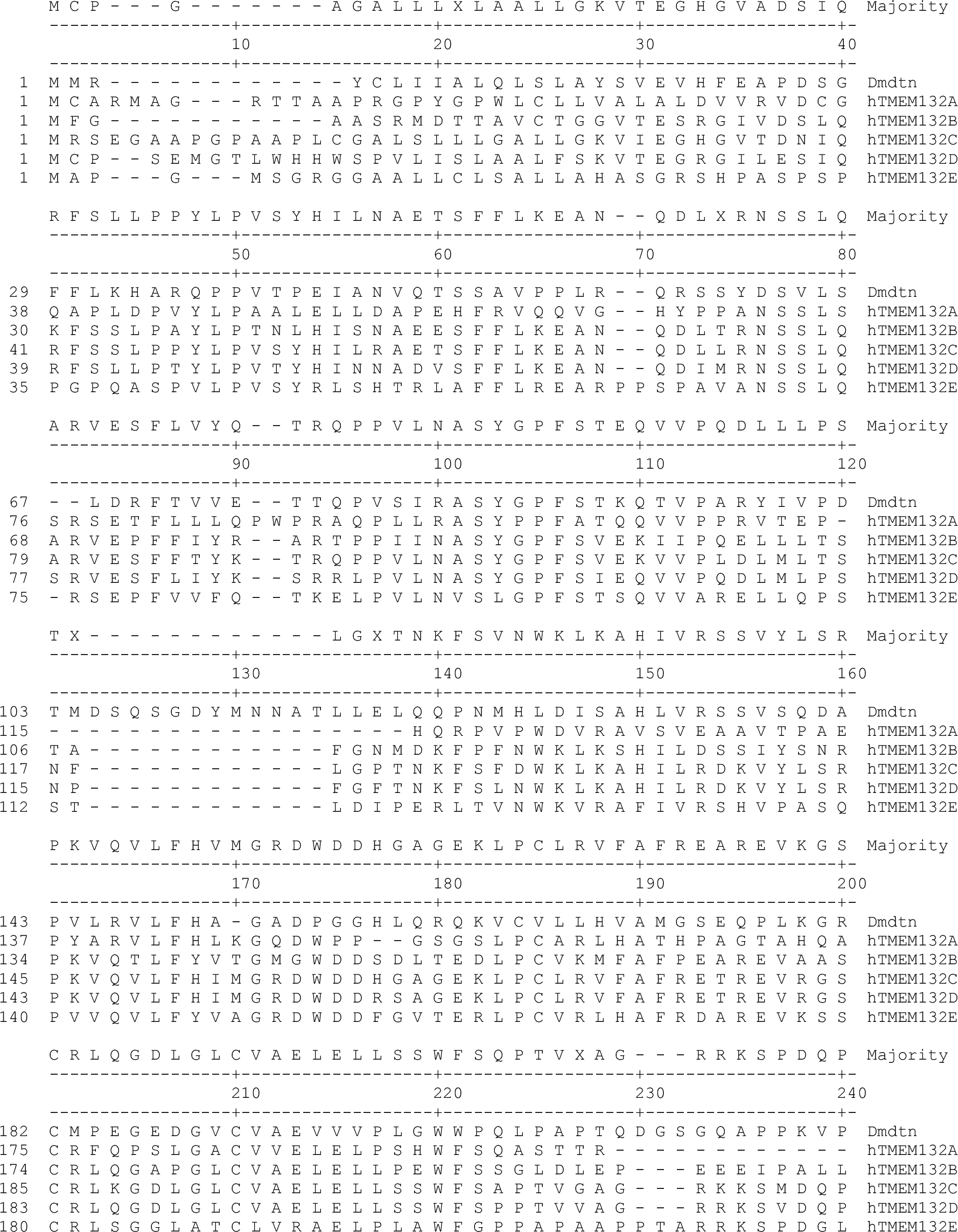

Supplement: S1 Fig — All peptide sequences were downloaded from http://ensembl.org. The multiple alignment was performed using Lasergene DNAStar with the Clustal W method. Dash lines in individual protein sequence indicate gaps, whereas dash lines in the Majority sequence indicate lack of consensus residue at corresponding position. (ZIP) [file pone.0245454.s001.zip › S1 Fig page1.tif]

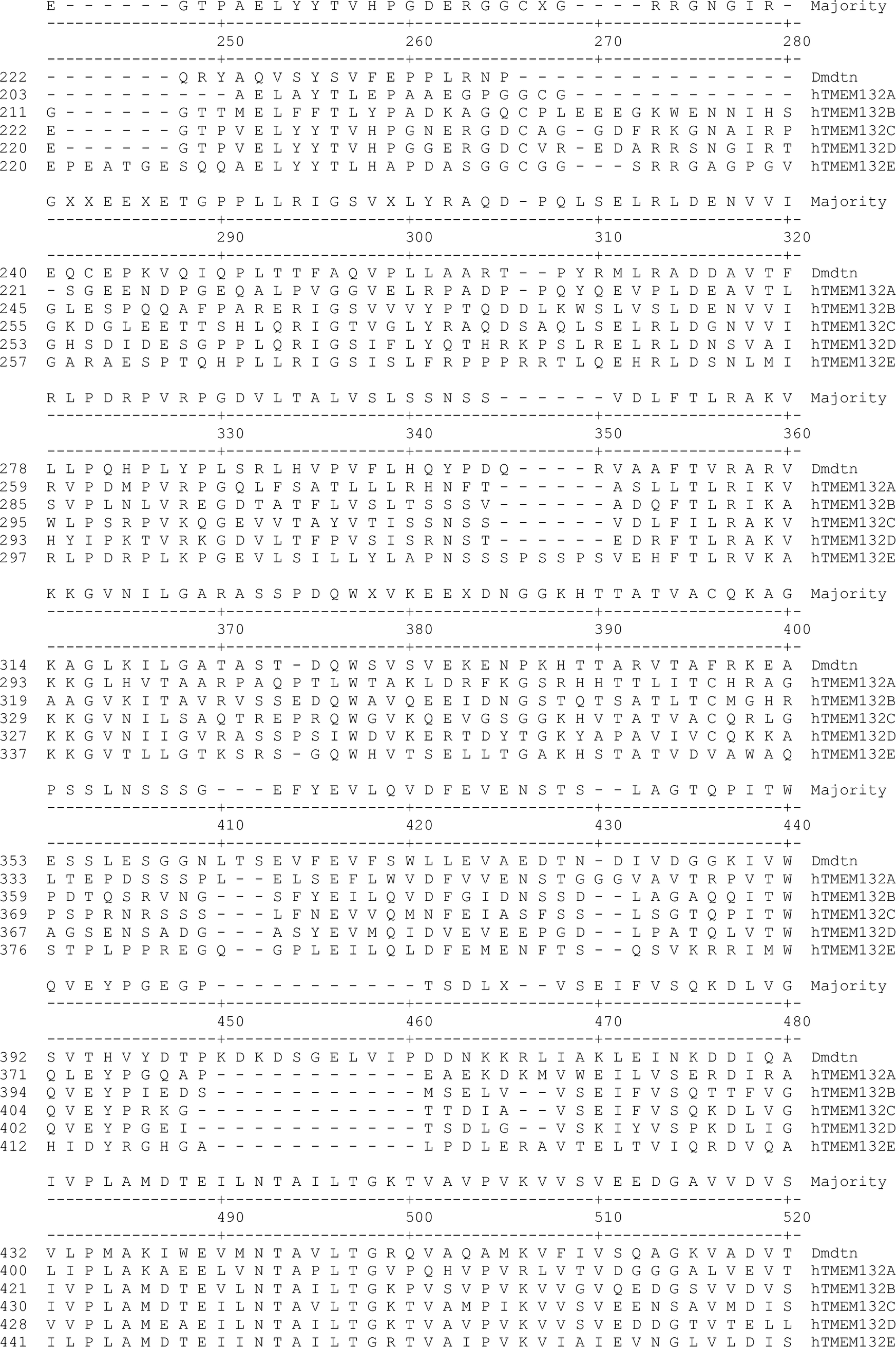

Supplement: S1 Fig — All peptide sequences were downloaded from http://ensembl.org. The multiple alignment was performed using Lasergene DNAStar with the Clustal W method. Dash lines in individual protein sequence indicate gaps, whereas dash lines in the Majority sequence indicate lack of consensus residue at corresponding position. (ZIP) [file pone.0245454.s001.zip › S1 Fig page2.tif]

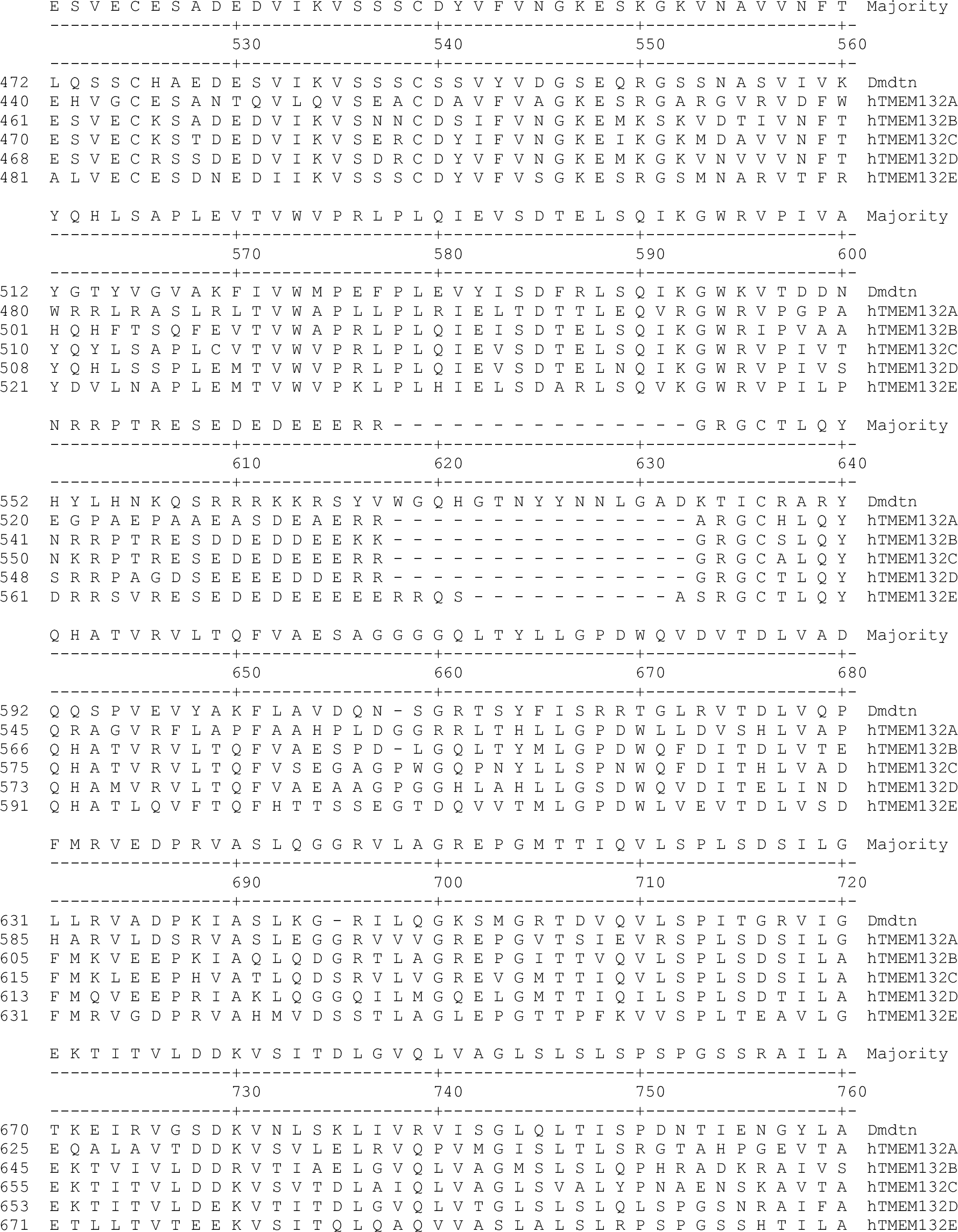

Supplement: S1 Fig — All peptide sequences were downloaded from http://ensembl.org. The multiple alignment was performed using Lasergene DNAStar with the Clustal W method. Dash lines in individual protein sequence indicate gaps, whereas dash lines in the Majority sequence indicate lack of consensus residue at corresponding position. (ZIP) [file pone.0245454.s001.zip › S1 Fig page3.tif]

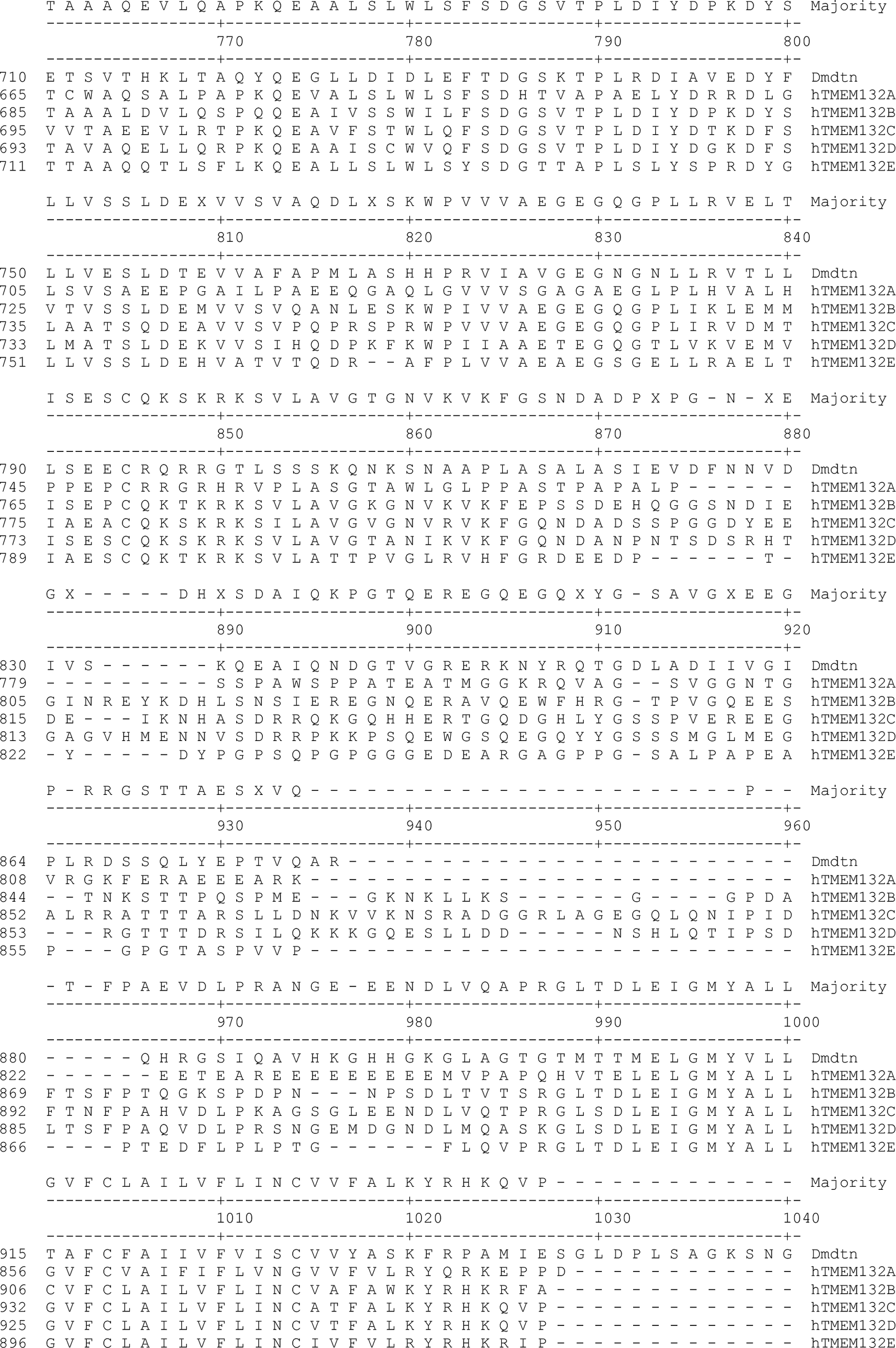

Supplement: S1 Fig — All peptide sequences were downloaded from http://ensembl.org. The multiple alignment was performed using Lasergene DNAStar with the Clustal W method. Dash lines in individual protein sequence indicate gaps, whereas dash lines in the Majority sequence indicate lack of consensus residue at corresponding position. (ZIP) [file pone.0245454.s001.zip › S1 Fig page4.tif]

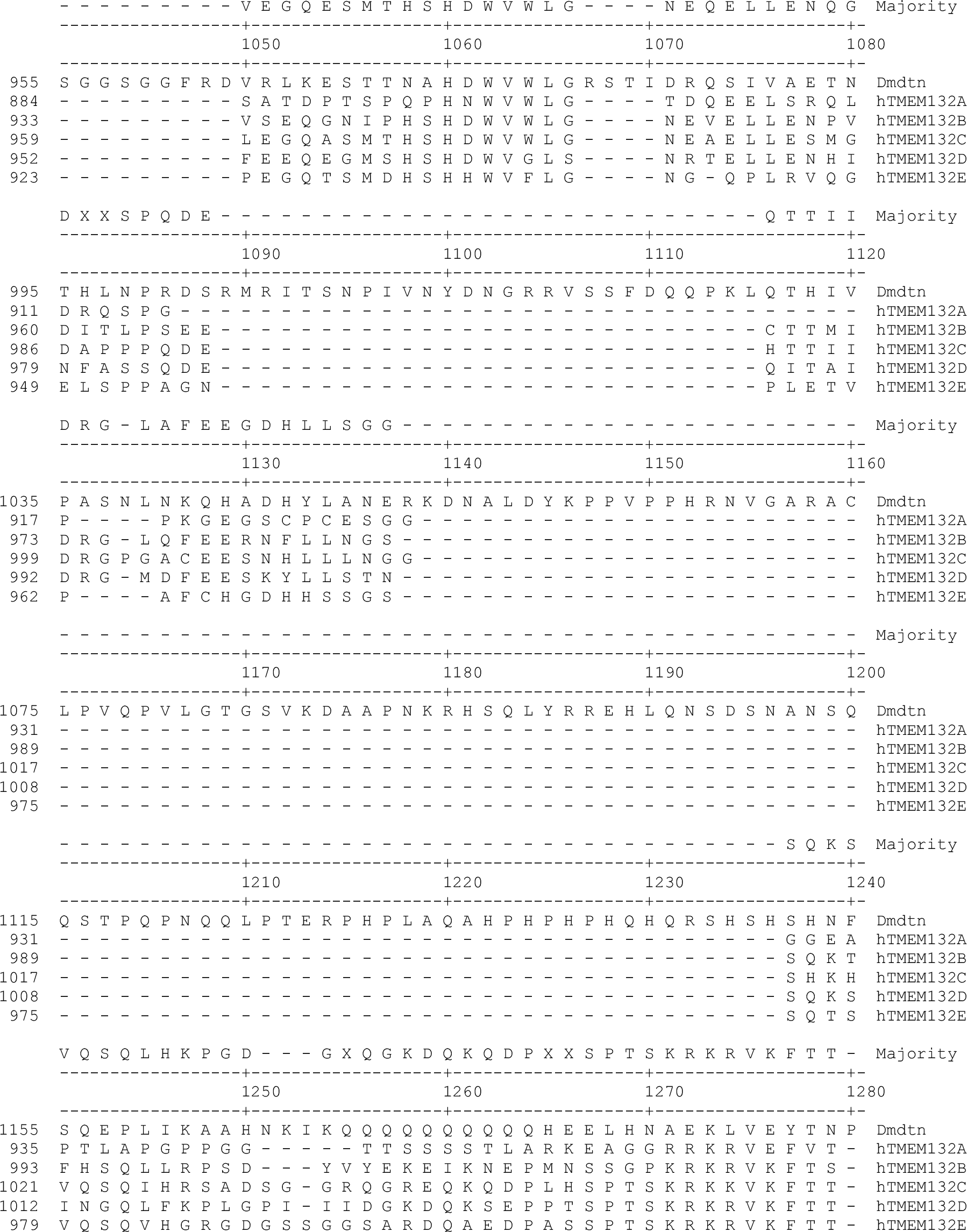

Supplement: S1 Fig — All peptide sequences were downloaded from http://ensembl.org. The multiple alignment was performed using Lasergene DNAStar with the Clustal W method. Dash lines in individual protein sequence indicate gaps, whereas dash lines in the Majority sequence indicate lack of consensus residue at corresponding position. (ZIP) [file pone.0245454.s001.zip › S1 Fig page5.tif]

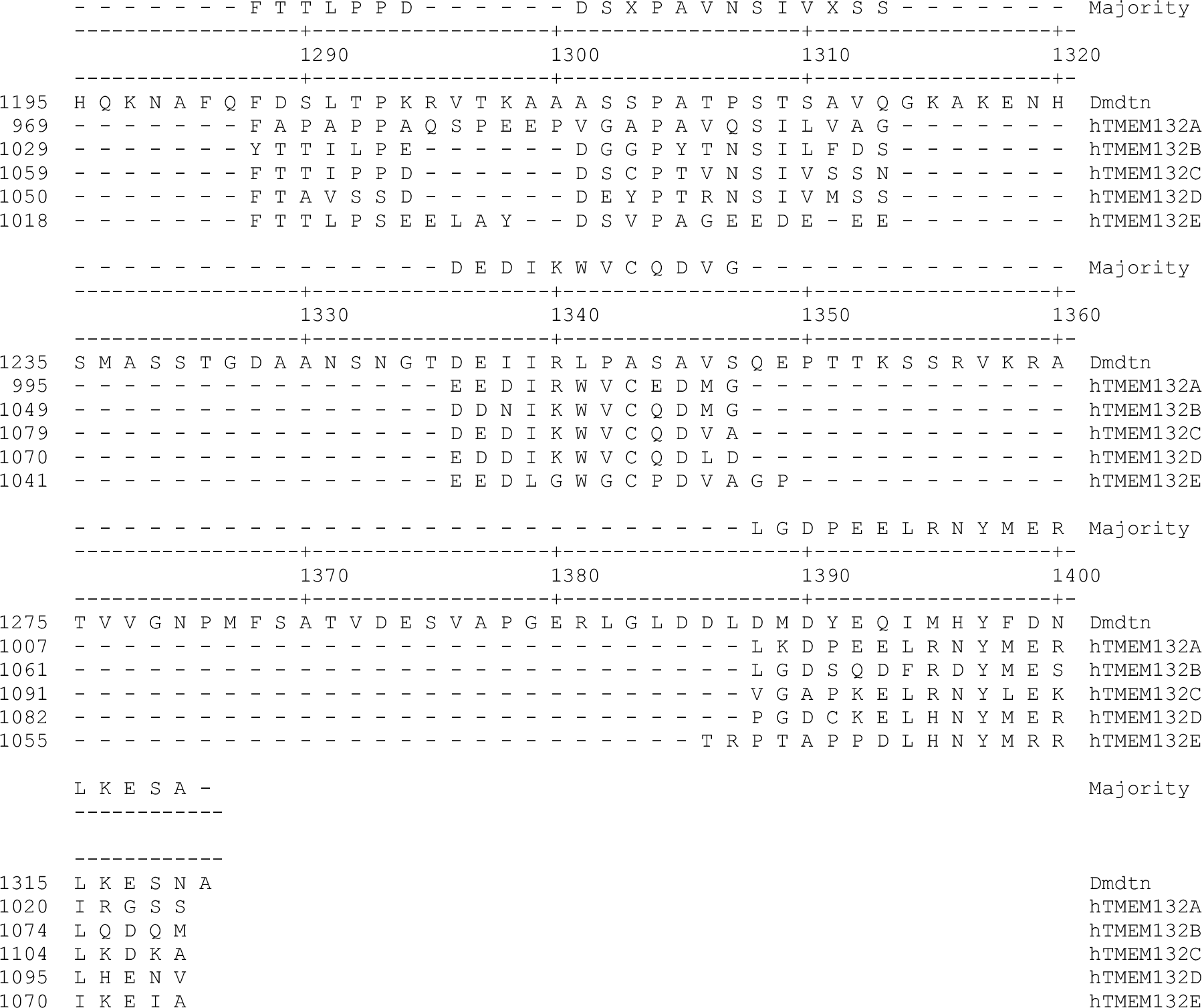

Supplement: S1 Fig — All peptide sequences were downloaded from http://ensembl.org. The multiple alignment was performed using Lasergene DNAStar with the Clustal W method. Dash lines in individual protein sequence indicate gaps, whereas dash lines in the Majority sequence indicate lack of consensus residue at corresponding position. (ZIP) [file pone.0245454.s001.zip › S1 Fig page6.tif]

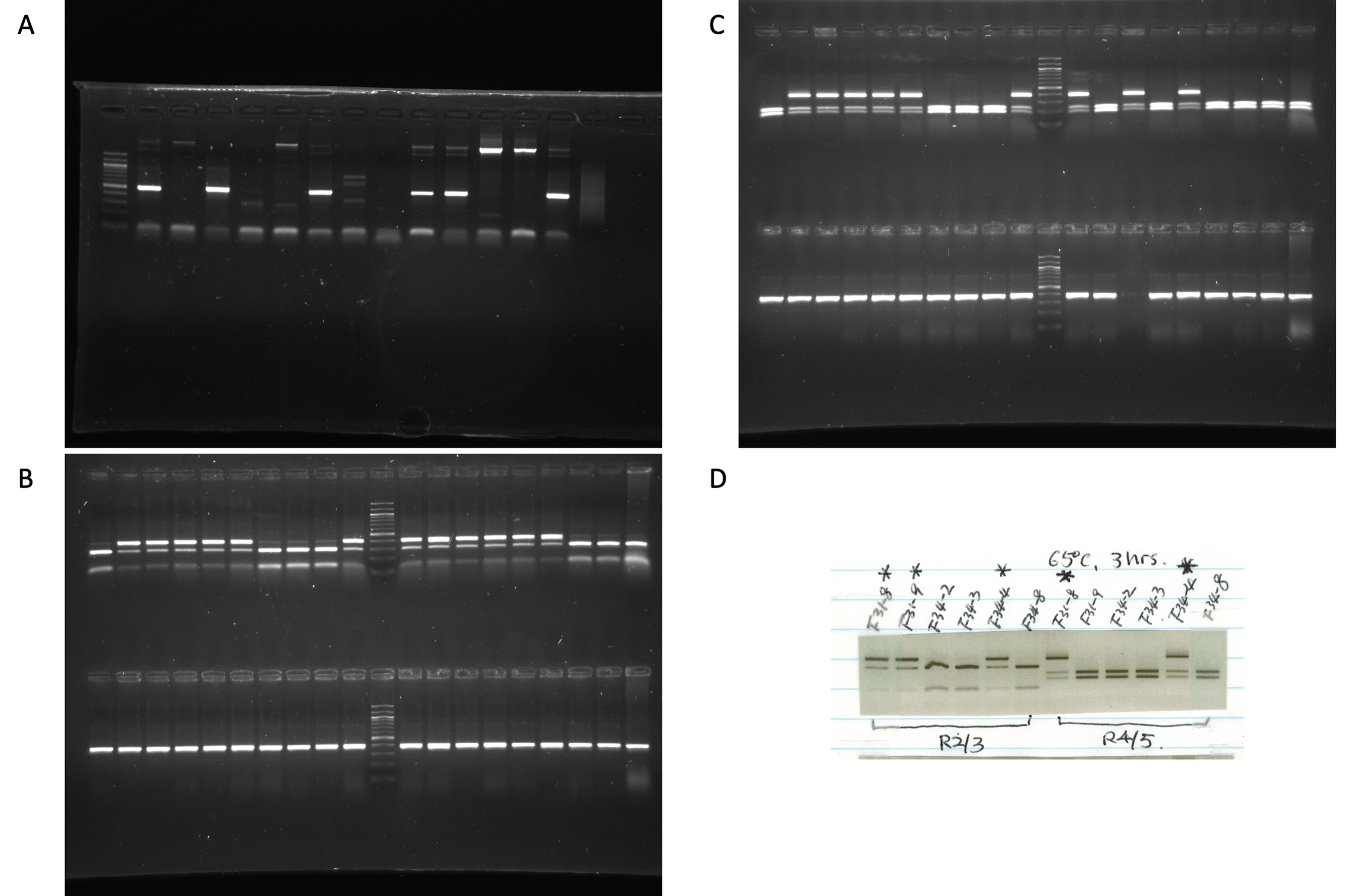

Supplement: S2 Fig — (A) Whole gel photo for Fig 2B. (B) Whole gel photo for Fig 2C. (C) Whole gel photo for Fig 2D. (D) Scanned gel image from the original notebook. The digital file for the whole gel photo was not saved. (TIF) [file pone.0245454.s002.tif]
